# Supplementary material for: Robust immune cell infiltration and macrophage senescence occur within a week of recovery after limb immobilization in older adult skeletal muscle
Source: J Physiol. 2026 Mar 27;604(8):3379–95. doi: 10.1113/JP290346 (PMC13082186; doi:10.1113/JP290346)
Supplement: Supplementary file 2 — Supporting Information [file TJP-604-3379-s001.docx]

**APPENDIX**

Tables A1-A4.

Figures A1-A7.

Supplemental Methodology.

**Table A1. Physical Activity and Lean Mass Changes**

|  | **YA** | **% Change**  **From Pre** | **OA** | **% Change**  **From Pre** |
| --- | --- | --- | --- | --- |
| **PA (Steps/d)^a,b^** |  |  |  |  |
| Pre | 9941 ± 3398***** |  | 5685 ± 2300 |  |
| Post-Immob | 4529 ± 2165***** | -51 ± 27 | 2648 ± 1800 | -51 ± 32 |
| 7d-Rec | 8371 ± 2810***** | -12 ± 27 | 5202 ± 2324 | -6 ± 32 |
| **L Leg LM (kg)^a,b^** |  |  |  |  |
| Pre | 8.9 ± 1.9 |  | 7.2 ± 1.7 |  |
| Post-Immob | 8.6 ± 1.8 | -4 ± 2 | 7.0 ± 1.7 | -3 ± 5 |
| 7d-Rec | 8.7 ± 1.9 | -3 ± 2 | 7.2 ± 1.8 | -1 ± 4 |
| **L Quad LM (kg)^a,b^** |  |  |  |  |
| Pre | 7.6 ± 1.5 |  | 6.3 ± 1.3 |  |
| Post-Immob | 7.3 ± 1.4 | -3 ± 4 | 6.1 ± 1.3 | -2 ± 4 |
| 7d-Rec | 7.4 ± 1.4 | -2 ± 4 | 6.2 ± 1.3 | -1 ± 5 |
| **R + L Leg LM (kg)^a,b^** |  |  |  |  |
| Pre | 18.0 ± 3.7 |  | 14.6 ± 3.4 |  |
| Post-Immob | 17.6 ± 3.5 | -2 ± 2 | 14.1 ± 3.4 | -4 ± 4 |
| 7d-Rec | 17.7 ± 3.7 | -2 ± 2 | 14.5 ± 3.5 | -1 ± 3 |
| **R+L Quad LM (kg)^a,b^** |  |  |  |  |
| Pre | 15.2 ± 2.9 |  | 12.5 ± 2.6 |  |
| Post-Immob | 14.8 ± 2.8 | -3 ± 3 | 12.2 ± 2.4 | -3 ± 3 |
| 7d-Rec | 14.9 ± 2.9 | -2 ± 3 | 12.4 ± 2.5 | -1 ± 4 |

Data presented as Mean±SD. ^a^ = time main effect. ^b^ = age main effect. *vs old counterpart. Immob,

immobilization; L, left leg; LM, lean mass; OA, older adults; Quad, quadriceps; R, right; YA, young adults.

|  | **YM** | **OM** |  | **YF** | **OF** |
| --- | --- | --- | --- | --- | --- |
| **PA (steps/d)^a,b^** |  |  | **PA (steps/d)^a,b^** |  |  |
| Pre | 8281±2384 | 5147±1926 | Pre | 11602±3556 | 6223±2623 |
| Post-Immob | 3840±2031 | 2910±1904 | Post-Immob | 5217±2184 | 2385±1761 |
| 7d-Rec | 8057±2923 | 5214±2215 | 7d-Rec | 8686±2929 | 5189±2563 |
| **L Leg LM (kg)^a,b^** |  |  | **L Leg LM (kg)^a,b^** |  |  |
| Pre | 10.3±1.0 | 8.5±1.1 | Pre | 7.2±1.1 | 5.8±0.9 |
| Post-Immob | 9.9±0.9 | 8.3±1.1 | Post-Immob | 6.9±1.0 | 5.5±0.9 |
| 7d-Rec | 10.0±1.1 | 8.5±1.1 | 7d-Rec | 7.0±1.1 | 5.7±1.0 |
| **L Quad LM (kg)^a,b^** |  |  | **L Quad LM (kg)^b^** |  |  |
| Pre | 8.6±0.8 | 7.3±0.8 | Pre | 6.3±1.0 | 5.1±0.7 |
| Post-Immob | 8.3±0.8 | 7.1±0.7 | Post-Immob | 6.1±1.0 | 5.0±0.7 |
| 7d-Rec | 8.4±0.7 | 7.1±0.7 | 7d-Rec | 6.1±0.9 | 5.1±0.8 |
| **R+L Leg LM (kg)^a,b^** |  |  | **R+L Leg LM (kg)^a,b^** |  |  |
| Pre | 20.8±2.0 | 17.2±2.0 | Pre | 14.6±2.1 | 11.7±1.8 |
| Post-Immob | 20.2±1.8 | 16.7±2.1 | Post-Immob | 14.4±2.0 | 11.2±1.8 |
| 7d-Rec | 20.4±2.2 | 17.1±2.2 | 7d-Rec | 14.4±2.0 | 11.5±2.1 |
| **R+L Quad LM (kg)^a,b^** |  |  | **R+L Quad LM (kg)^a,b^** |  |  |
| Pre | 17.3±1.7 | 14.5±1.4 | Pre | 12.6±2.0 | 10.3±1.5 |
| Post-Immob | 16.7±1.6 | 14.0±1.3 | Post-Immob | 12.2±1.9 | 10.1±1.4 |
| 7d-Rec | 17.0±1.6 | 14.2±1.3 | 7d-Rec | 12.2±1.8 | 10.3±1.7 |

**Table A2. Physical Activity and Lean Mass Changes by Sex**

Data presented as Mean±SD.^a^ = time main effect. ^b^ = age main effect. *P<0.05 vs old counterpart. Immob, immobilization; L, left leg; LM, lean mass; OF, older females; OM, older males; Quad, quadriceps; R, right; YF, young females; YM, young males.

**Table A3. Myofiber Characteristics**

|  | **YA** | **Δ From Pre** | **OA** | **Δ From Pre** |
| --- | --- | --- | --- | --- |
| **MHC I Composition (%)** |  |  |  |  |
| Pre | 44 ± 14 |  | 51 ± 18 |  |
| Post-Immob | 41 ± 16 | -2 ± 20 | 50 ± 20 | -1 ± 11 |
| 2d-Rec | 38 ± 16 | -6 ± 16 | 53 ± 15 | 2 ± 18 |
| 7d-Rec | 43 ± 14 | -1 ± 12 | 48 ± 19 | -2 ± 16 |
| **MHC I Fiber CSA (µm^2^)^a^** |  |  |  |  |
| Pre | 4101 ± 1262 |  | 3677 ± 1141 |  |
| Post-Immob | 3745 ± 1199 | -357 ± 804 | 3241 ± 871 | -436 ± 611 |
| 2d-Rec | 3832 ± 1115 | -378 ± 1033 | 3285 ± 935 | -393 ± 684 |
| 7d-Rec | 3994 ± 1458 | -108 ± 1152 | 3381 ± 948 | -364 ± 923 |
| **MHC IIA Fiber CSA (µm^2^)^b^** |  |  |  |  |
| Pre | 4542 ± 1678 |  | 2750 ± 1139 |  |
| Post-Immob | 4197 ± 1674 | -345 ± 1102 | 2283 ± 685 | -467 ± 896 |
| 2d-Rec | 4135 ± 1709 | -506 ± 1221 | 2481 ± 956 | -270 ± 480 |
| 7d-Rec | 4314 ± 2188 | -228 ± 1443 | 2312 ± 782 | -472 ± 815 |
| **MN Number (MN/f)** |  |  |  |  |
| Pre | 4.0 ± 0.6 |  | 3.6 ± 0.6 |  |
| Post-Immob | 3.8 ± 0.6 | -0.1 ± 0.6 | 3.8 ± 0.6 | 0.2 ± 0.3 |
| 2d-Rec | 3.7 ± 0.5 | -0.2 ± 0.7***** | 4.0 ± 0.5 | 0.4 ± 0.5 |
| 7d-Rec | 4.0 ± 0.8 | 0.1 ± 0.8 | 3.8 ± 0.8 | 0.3 ± 0.7 |
| **MND (µm^2^/MN)^a,b^** |  |  |  |  |
| Pre | 1243 ± 199 |  | 1116 ± 150 |  |
| Post-Immob | 1158 ± 173 | -88 ± 199 | 1016 ± 187 | -100 ± 135 |
| 2d-Rec | 1219 ± 204 | -28 ± 214 | 979 ± 140 | -137 ± 148 |
| 7d-Rec | 1125 ± 218 | -113 ± 239 | 980 ± 160 | -146 ± 179 |

Data presented as Mean±SD. ^a^ = time main effect. ^b^ = age main effect. *P<0.05 vs OA. *P<0.05 vs OA. Immob, immobilization; MHC,

myosin heavy chain; MN, myonuclei; MND, myonuclear domain; OA, older adults; YA, young adults.

**Table A4. Myofiber Characteristics by Sex**

|  | **YM** | **OM** |  | **YF** | **OF** |
| --- | --- | --- | --- | --- | --- |
| **MHC I Composition (%)** |  |  | **MHC I Composition (%)** |  |  |
| Pre | 43±16 | 50±18 | Pre | 45±12 | 51±19 |
| Post-Immob | 37±11 | 50±23 | Post-Immob | 47±19 | 49±19 |
| 2d-Rec | 34±15 | 53±15 | 2d-Rec | 43±17 | 52±17 |
| 7d-Rec | 43±16 | 49±22 | 7d-Rec | 43±12 | 47±17 |
| **MHC I Fiber CSA (µm^2^)** |  |  | **MHC I Fiber CSA (µm^2^)^a^** |  |  |
| Pre | 4362±1345 | 4081±1068 | Pre | 3775±1151 | 3273±1123 |
| Post-Immob | 4245±1214 | 3402±1021 | Post-Immob | 3120±888 | 3079±714 |
| 2d-Rec | 4529±881 | 3639±862 | 2d-Rec | 3047±791 | 2931±914 |
| 7d-Rec | 4681±1549 | 3769±1181 | 7d-Rec | 3135±749 | 3035±541 |
| **MHC IIA Fiber CSA (µm^2^)^b^** |  |  | **MHC IIA Fiber CSA (µm^2^)^a,b,c^** |  |  |
| Pre | 5016±2039 | 3472±1149 | Pre | 3948±877* | 2028±516 |
| Post-Immob | 4858±1827 | 2733±607 | Post-Immob | 3370±1055* | 1834±419 |
| 2d-Rec | 5387±1375 | 3077±935 | 2d-Rec | 2726±476* | 1884±518 |
| 7d-Rec | 5576±2174 | 2678±937 | 7d-Rec | 2736±657^ | 1987±451 |
| **MN Number (MN/f)** |  |  | **MN Number (MN/f)** |  |  |
| Pre | 4.2±0.6 | 4.1±0.5 | Pre | 3.7±0.6 | 3.1±0.3 |
| Post-Immob | 4.0±0.5 | 4.2±0.5 | Post-Immob | 3.6±0.7 | 3.5±0.5 |
| 2d-Rec | 3.9±0.4 | 4.3±0.4 | 2d-Rec | 3.5±0.6 | 3.7±0.5 |
| 7d-Rec | 4.5±0.7 | 4.4±0.7 | 7d-Rec | 3.5±0.7 | 3.4±0.6 |
| **MND (µm^2^/MN)^a,b^** |  |  | **MND (µm^2^/MN)^b^** |  |  |
| Pre | 1312±174 | 1164±117 | Pre | 1148±173 | 1069±171 |
| Post-Immob | 1165±199 | 1021±127 | Post-Immob | 1131±116 | 1011±242 |
| 2d-Rec | 1233±205 | 1056±122 | 2d-Rec | 1195±202 | 902±117 |
| 7d-Rec | 1118±222 | 992±155 | 7d-Rec | 1136±198 | 967±173 |

Data presented as Mean±SD. ^a^ = time main effect. ^b^ = age main effect. ^c^ = time x age interaction. *P<0.05 vs old counterpart. ^P<0.10 vs old counterpart. Immob, immobilization; MHC, myosin heavy chain; MN, myonuclei; MND, myonuclear domain; OF, older females; OM, older males; YF, young females; YM, young males.

(B)

(A)


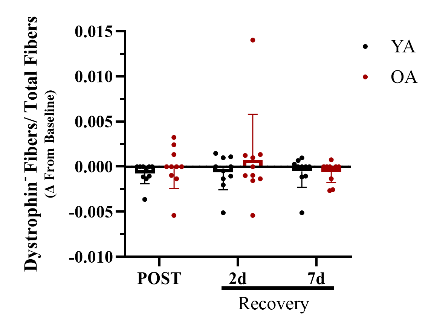

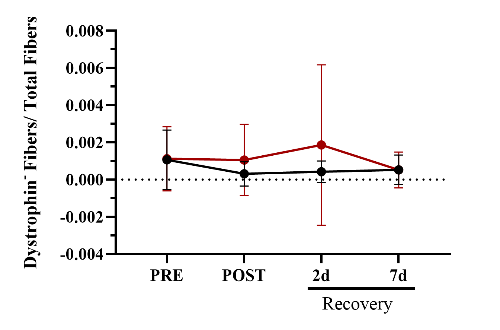


(A)

(A)


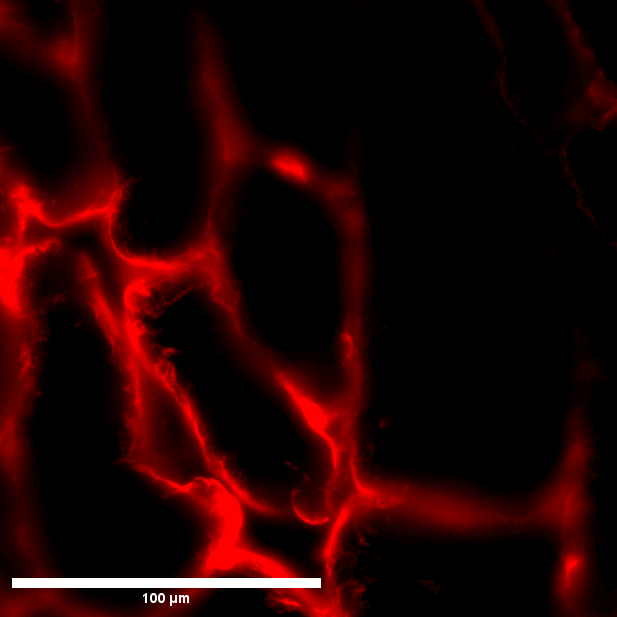


*


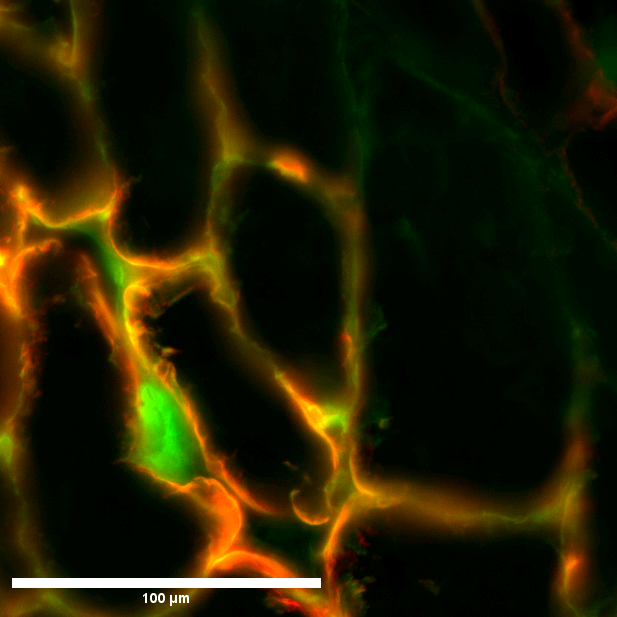


*

Laminin/Dystrophin


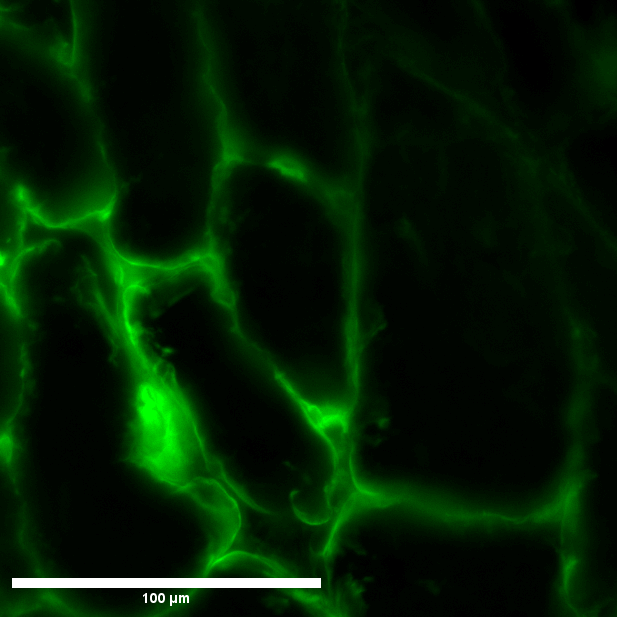


*

(C)

(A)

**7d-Recovery**

**Figure A1.** **Muscle Dystrophin.** (A) Number of fibers with missing dystrophin per total number of fibers (YA: n=10; OA: n=10). (B) Change in number of fibers with missing dystrophin per total number of fibers (Δ) from PRE (YA: n=10; OA: n=10). (C) Representative images to identify dystrophin^-^ fibers at 7d-recovery. *, Fibers with missing dystrophin. Data±SD

**
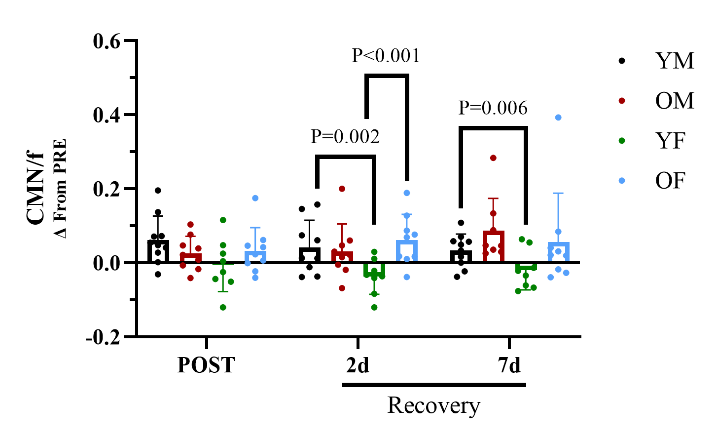
**


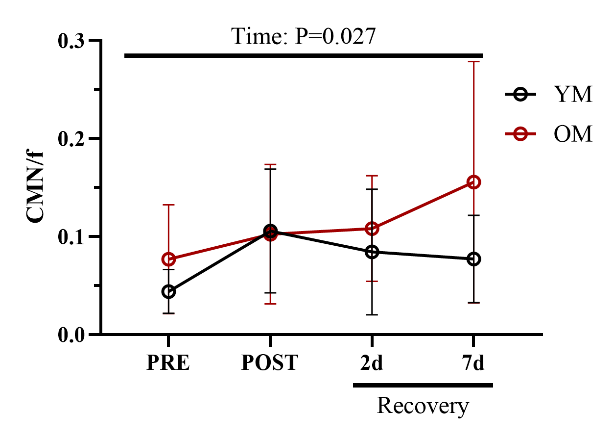


(A)

(A)

(C)

(A)


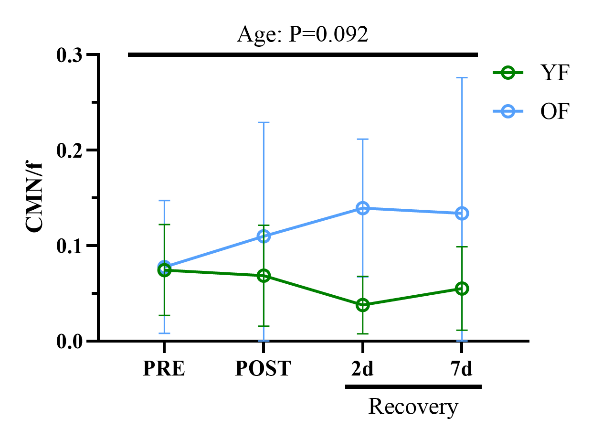


(B)

(A)


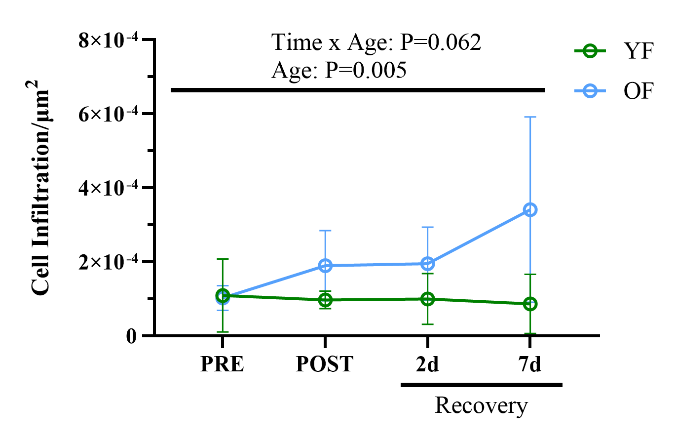


(E)

(A)


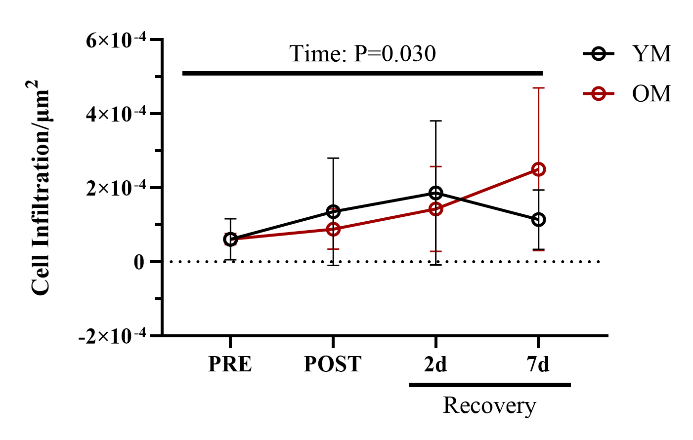


(D)

(A)


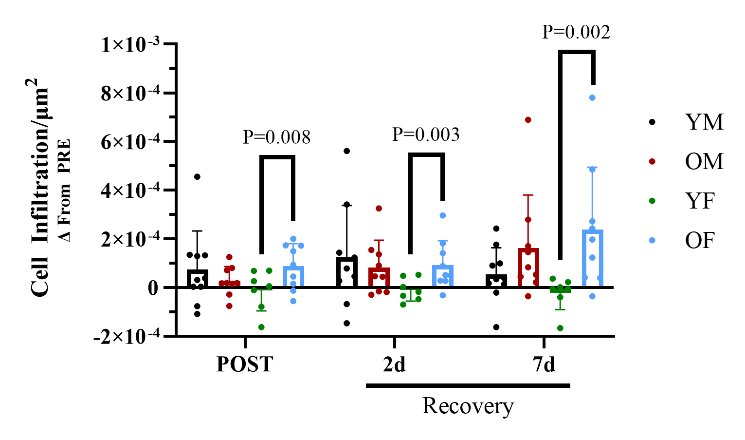


(F)

(A)


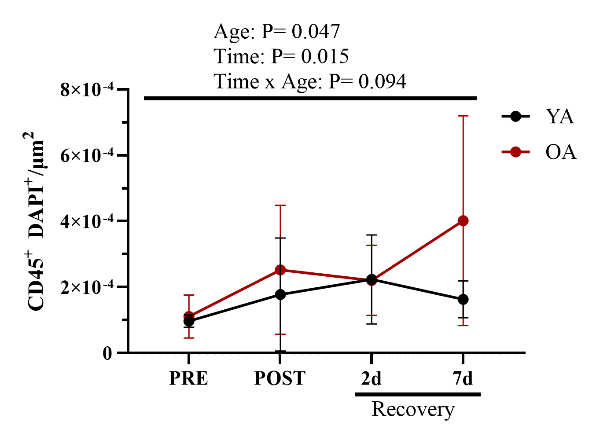


(G)

(A)


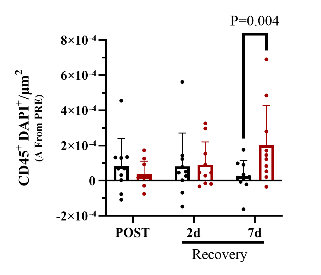


(H)

(A)

**7d-Recovery**


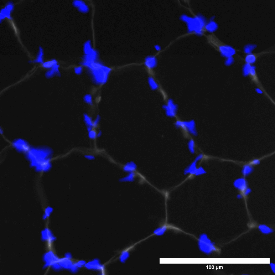

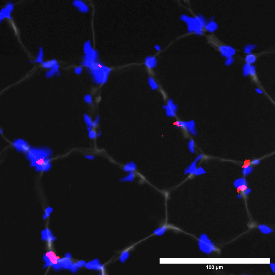


WGA/CD45/DAPI

(I)

(A)


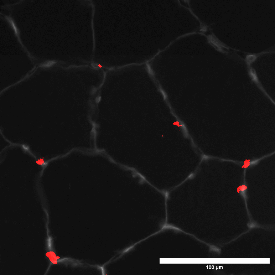


**Figure A2. Muscle Regeneration and Immune Cell Infiltration.**

**(A-C) Muscle Regeneration:** (A) Comparing young males and females in number of central myonuclei per fiber (PRE: YA: n=18 (10M/8F), OA: n=18 (9M/9F); POST: YA: n=18 (10M/8F), OA: n=18 (9M/9F); 2d-Recovery: YA: n=17 (9M/8F), OA: n=18 (9M/9F); 7d-Recovery: YA: n=18 (10M/8F), OA: n=17 (8M/9F). (B) Comparing older males and females in number of central myonuclei per fiber. (C) Comparing sex differences in number of central myonuclei per fiber (Δ) from PRE.

**(D-F) Immune Cell Infiltration:** (D) Comparing young males and females in immune cell infiltration (cell/µm^2^) (PRE: YA: n=18 (10M/8F), OA: n=18 (9M/9F); POST: YA: n=18 (10M/8), OA: n=18 (9M/9F); 2d-Recovery: YA: n=17 (9M/8), OA: n=18 (9M/9F); 7d-Recovery: YA: n=18 (10M/8F), OA: n=17 (8M/9F). (E) Comparing older males and females in cellular infiltration (cell/µm^2^). (F) Comparing sex differences in cellular infiltration (cell/µm^2^) (Δ) from PRE. (G) CD45^+^ DAPI^+^ per µm^2^ (All time points: YA: n=10 (5M/5F), OA: n=10 (5M/5F); (H) Change in CD45^+^ DAPI^+^ per µm^2^ (Δ) from PRE. (I) Representative images used to identify CD45^+^ cells at 7d-recovery.

Data±SD. OA, older adults; OF, older females; OM, older males; YA, young adults; YF, young females; YM, young males.

(A)

(A)

(B)

(A)


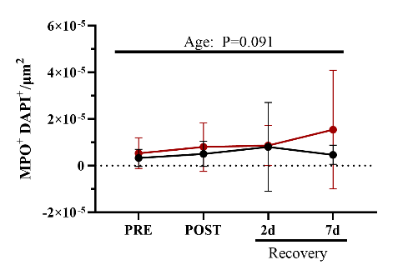

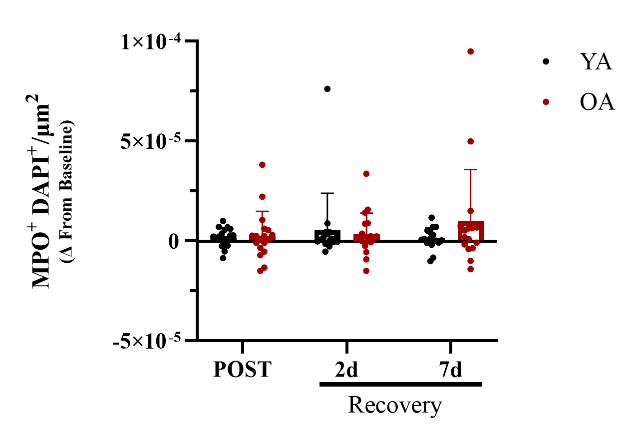

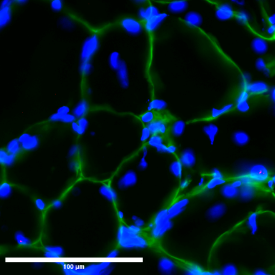


Laminin/MPO/DAPI


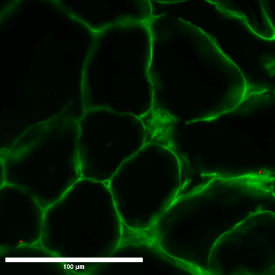

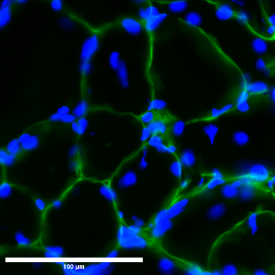


(C)

(A)

**7d-Recovery**


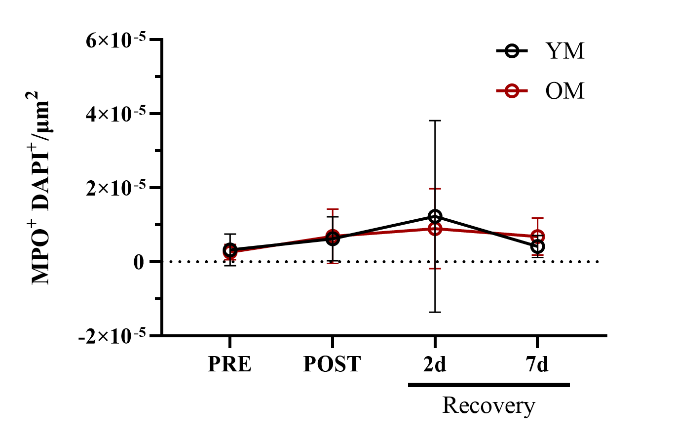


(D)

(A)


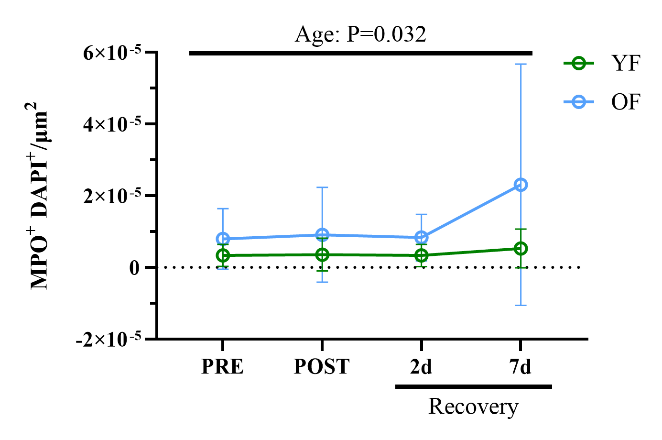


(E)

(A)


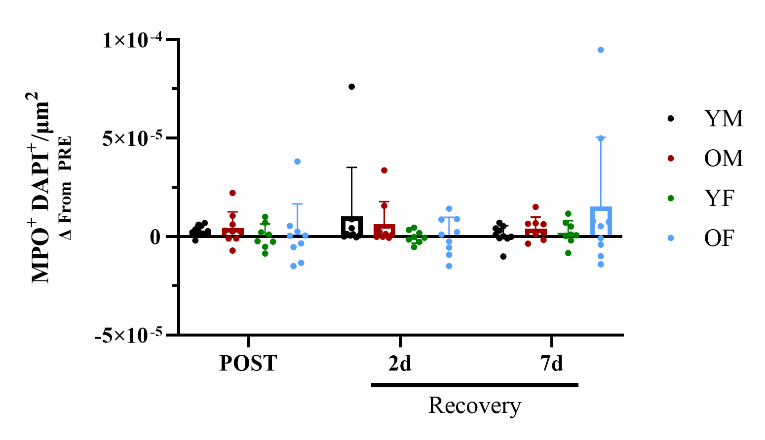


(F)

(A)


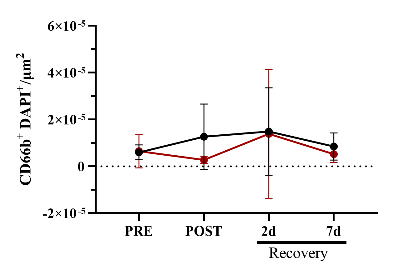


(G)

(A)


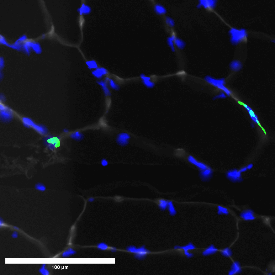

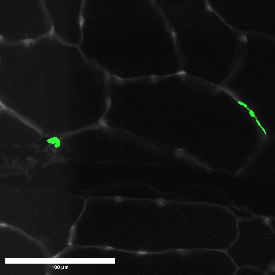

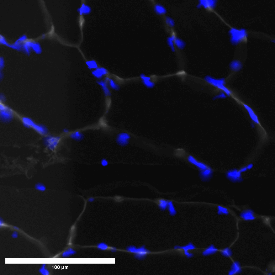


**7d-Recovery**

WGA/CD66b/DAPI

(I)

(A)


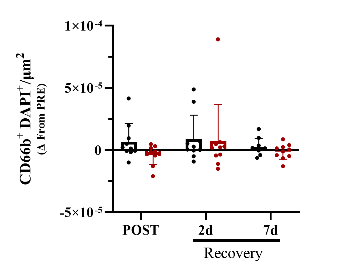


(H)

(A)

**Figure A3. Neutrophil Infiltration.** (A) Myeloperoxidase (MPO)^+^ DAPI^+^ per µm^2^ (PRE: YA: n=18 (10M/8F), OA: n=18 (9M/9F); POST: YA: n=18 (10M/8F), OA: n=18 (9M/9F); 2d-Recovery: YA: n=17 (9M/8F), OA: n=18 (9M/9F); 7d-Recovery: YA: n=18 (10M/8F), OA: n=17 (8M/9F). (B) Change in MPO^+^ DAPI^+^ per µm^2^ (Δ) from PRE. (C) Representative images used to identify MPO^+^ cells at 7d-recovery. (D) Comparing young males and females in MPO^+^ DAPI^+^ per µm^2^. (E) Comparing older males and females in MPO^+^ DAPI^+^ per µm^2^. (F) Comparing sex differences in change in MPO^+^ DAPI^+^ per µm^2^ (Δ). (G) CD66b^+^ DAPI^+^ per µm^2^ (All time points: YA: n=10 (5M/5F), OA: n=10 (5M/5F); (H) Change in CD66b^+^ DAPI^+^ per µm^2^ (Δ) from PRE. (I) Representative images used to identify CD66b^+^ cells at 7d-recovery.

Data±SD. OA, older adults; OF, older females; OM, older males; YA, young adults; YF, young females; YM, young males.

**
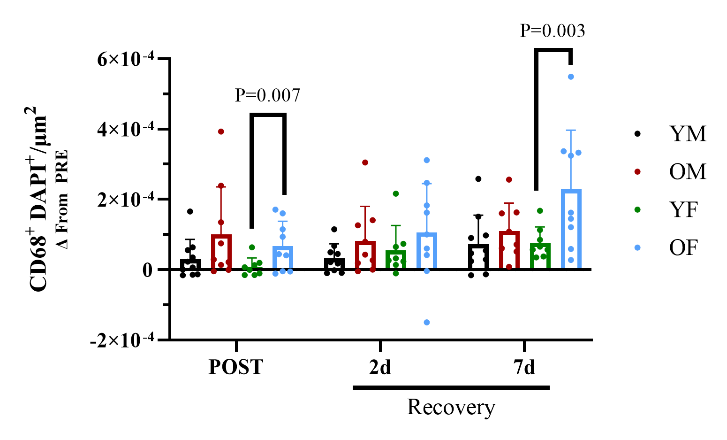

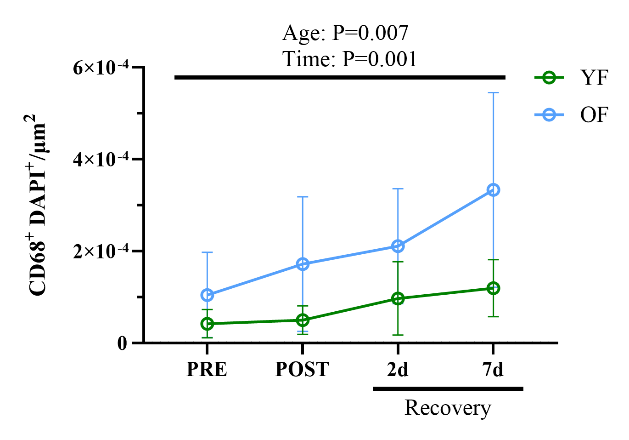
**


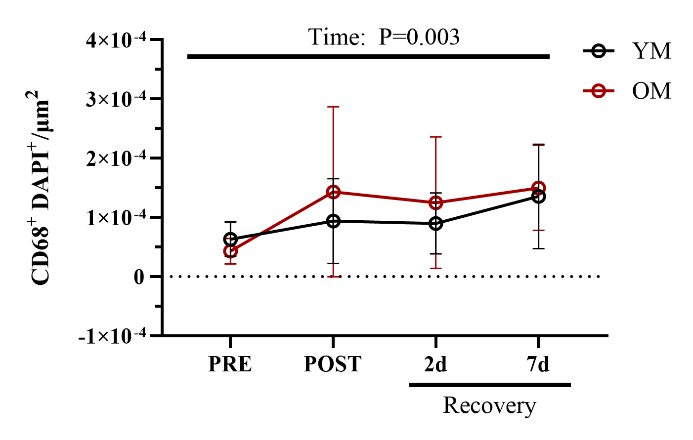


(A)

(A)

(C)

(A)

(B)

(A)


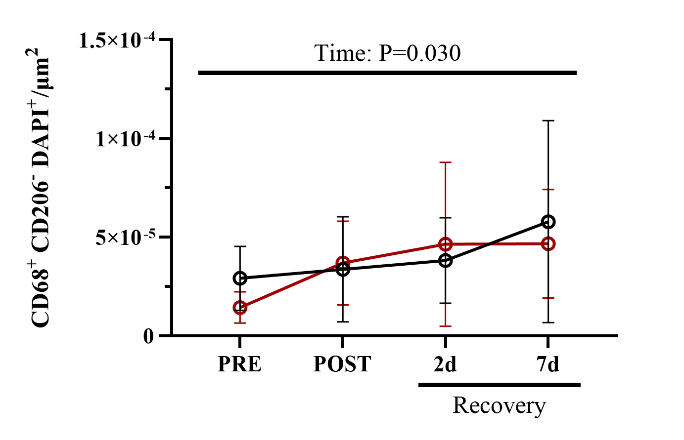


(D)

(A)


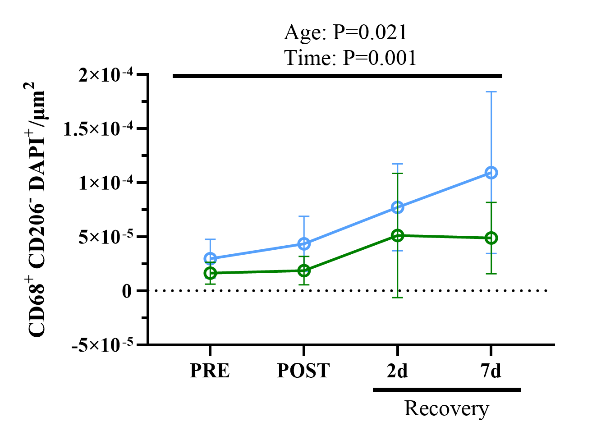


(E)

(A)

(F)

(A)


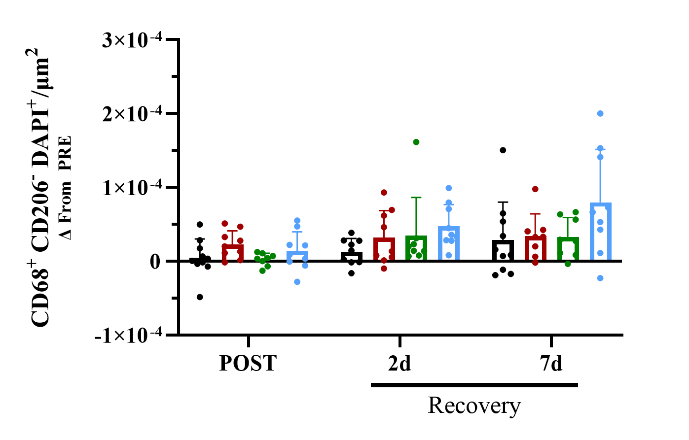


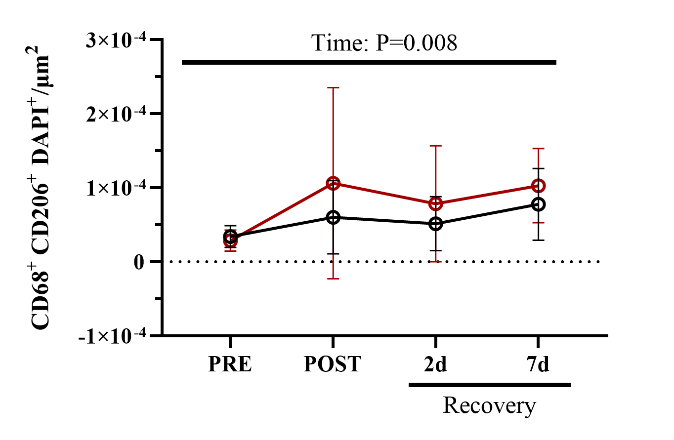


(G)

(A)

(I)

(A)

(H)

(A)


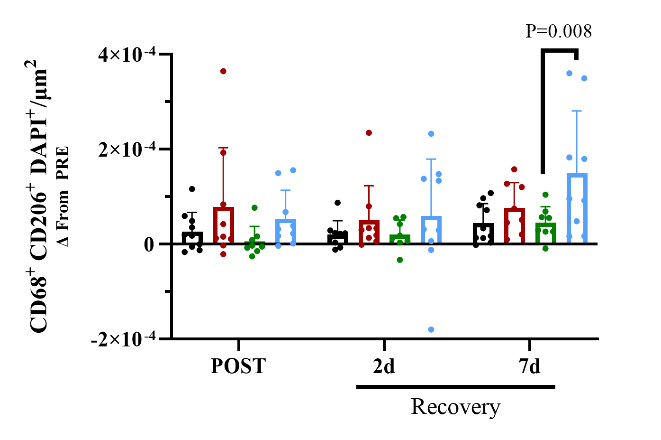

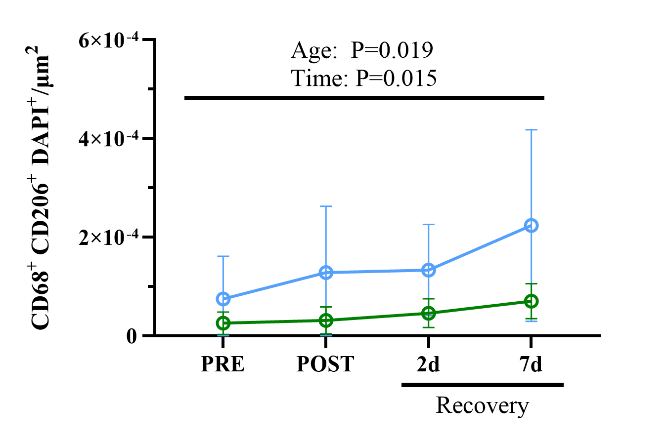


**Figure A4. Sex Differences in Infiltrating Macrophages.**

**(A-C) Total Macrophages (CD68^+^ DAPI^+^)**: (A) Comparing young males and females in total macrophages per µm^2^ (PRE: YA: n=18 (10M/8F), OA: n=18 (9M/9F); POST: YA: n=18 (10M/8F), OA: n=18 (9M/9F); 2d-Recovery: YA: n=17 (9M/8F), OA: n=18 (9M/9F); 7d-Recovery: YA: n=18 (10M/8F), OA: n=17 (8M/9F). (B) Comparing older males and females in total macrophages per µm^2^. (C) Comparing sex differences in number of total macrophages per µm^2^ (Δ) from PRE.

**(D-F) CD68^+^ CD206^-^ Macrophages**: (A) Comparing young males and females in CD68^+^ CD206^-^ macrophages per µm^2^. (B) Comparing older males and females in CD68^+^ CD206^-^ macrophages per µm^2^. (C) Comparing sex differences in number of CD68^+^ CD206^-^ macrophages per µm^2^ (Δ) from PRE.

**(G-I) CD68^+^ CD206^+^ Macrophages**: (A) Comparing young males and females in CD68^+^ CD206^+^ macrophages per µm^2^. (B) Comparing older males and females in CD68^+^ CD206^+^ macrophages per µm^2^. (C) Comparing sex differences in number of CD68^+^ CD206^+^ macrophages per µm^2^ (Δ) from PRE.

Data±SD. OA, older adults; OF, older females; OM, older males; YA, young adults; YF, young females; YM, young males


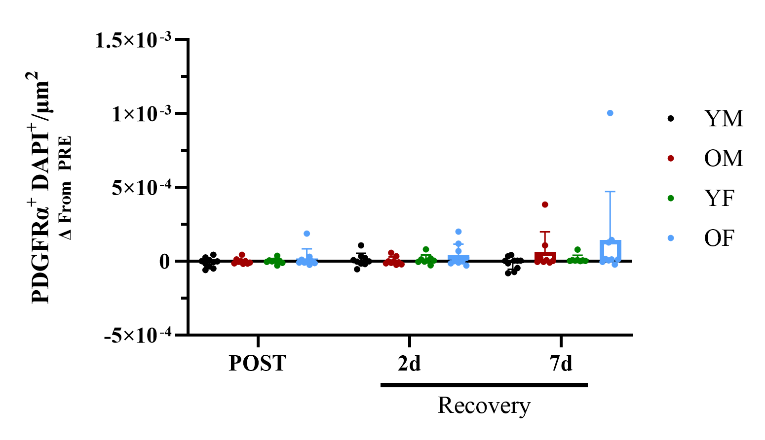

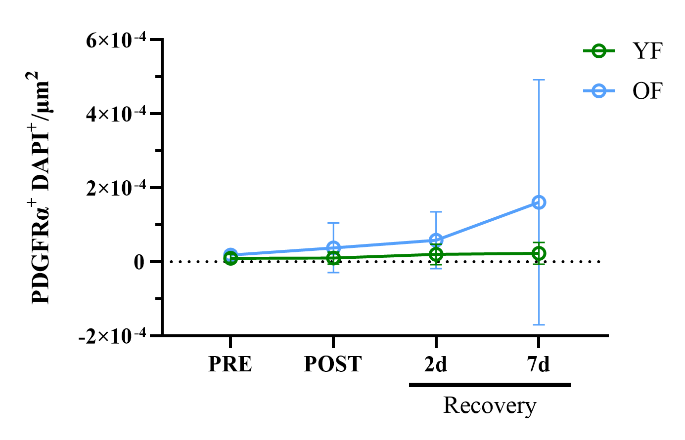

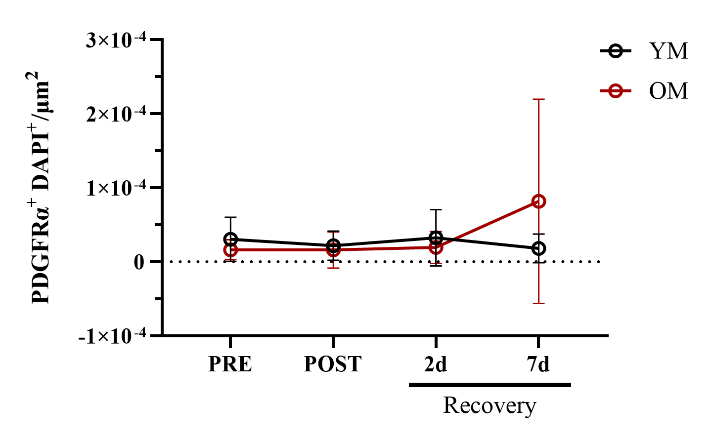


(C)

(A)

(B)

(A)

(A)

(A)

(F)

(A)

(E)

(A)

(D)

(A)


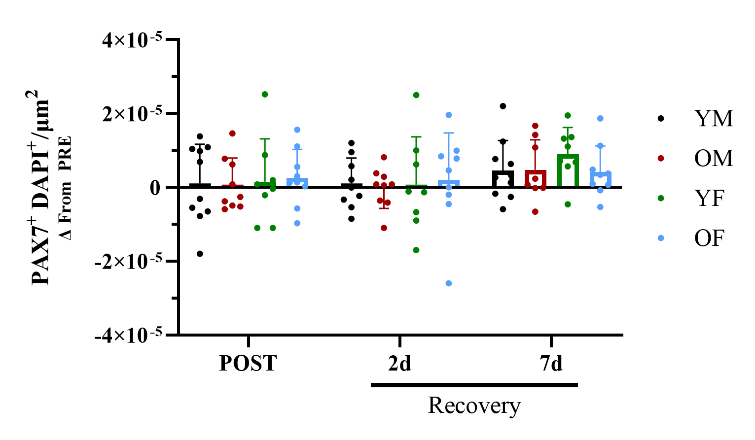

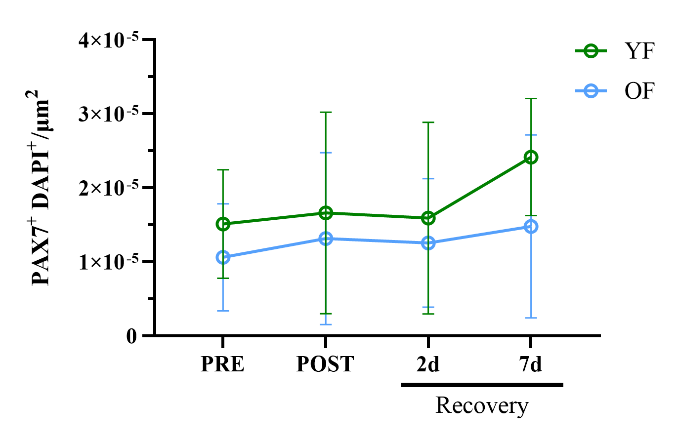

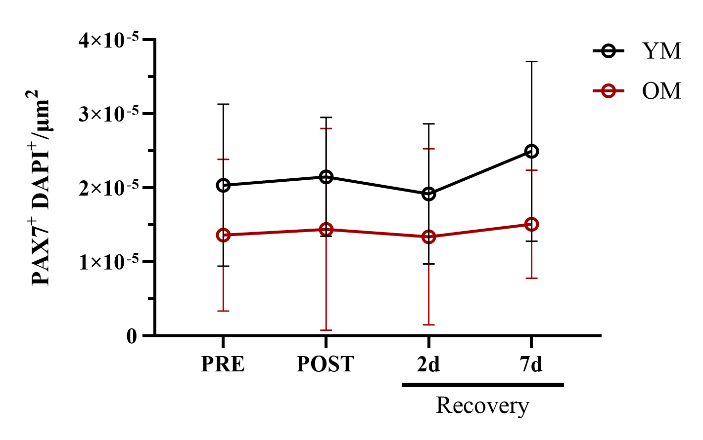


**Figure A5. Sex Differences in Fibro-Adipogenic Progenitor and Satellite Cell Content.**

**(A-C) Fibro-Adipongenic Progenitors (FAPs) (PDGFRα^+^ DAPI^+^)**: (A) Comparing young males and females in FAPs per µm^2^ (PRE: YA: n=18 (10M/8F), OA: n=18 (9M/9F); POST: YA: n=18 (10M/8F), OA: n=18 (9M/9F); 2d-Recovery: YA: n=17 (9M/8F), OA: n=18 (9M/9F); 7d-Recovery: YA: n=18 (10M/8F), OA: n=17 (8M/9F). (B) Comparing older males and females in FAPs per µm^2^. (C) Comparing sex differences in number of FAPs per µm^2^ (Δ) from PRE.

**(D-F) Satellite Cells (PAX7^+^ DAPI^+^)**: (A) Comparing young males and females in satellite cells per µm^2^ (PRE: YA: n=18 (10M/8F), OA: n=18 (9M/9F); POST: YA: n=18 (10M/8F), OA: n=18 (9M/9F); 2d-Recovery: YA: n=17 (9M/8F), OA: n=18 (9M/9F); 7d-Recovery: YA: n=18 (10M/8F), OA: n=17 (8M/9F).. (B) Comparing older males and females in satellite cells per µm^2^. (C) Comparing sex differences in number of satellite cells per µm^2^ (Δ) from PRE.

Data±SD. OA, older adults; OF, older females; OM, older males; YA, young adults; YF, young females; YM, young males


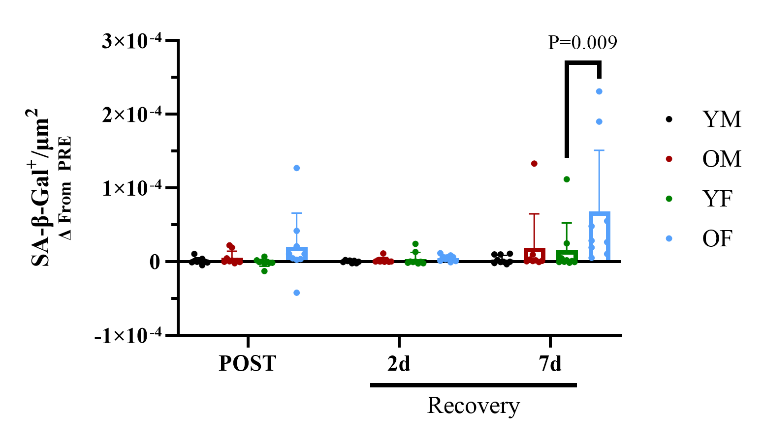


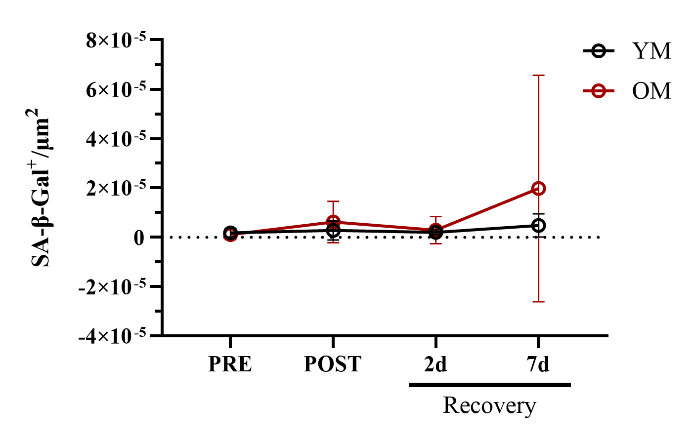


(A)

(A)


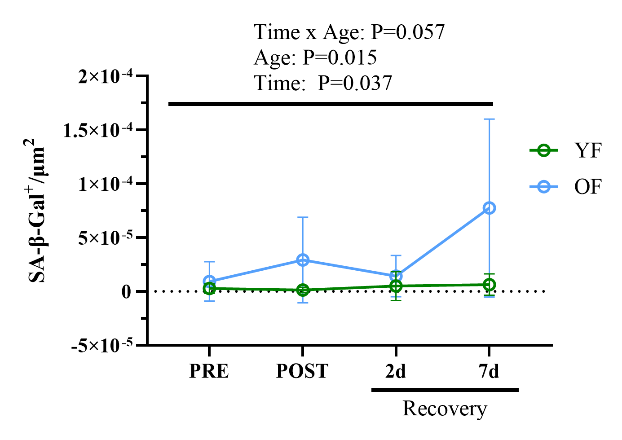


(B))

(A)

(C))

(A)


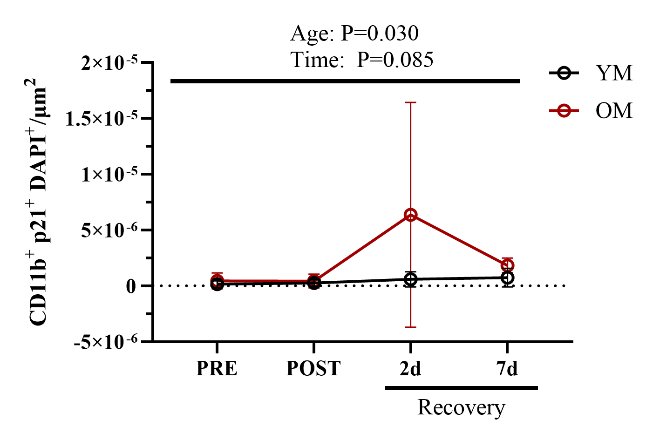


(D))

(A)


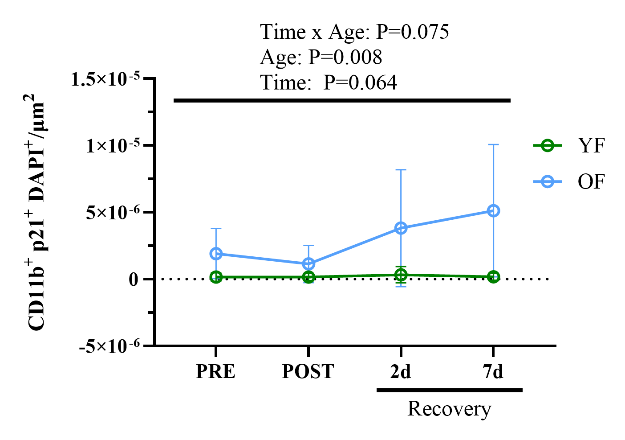


(E))

(A)

(F))

(A)


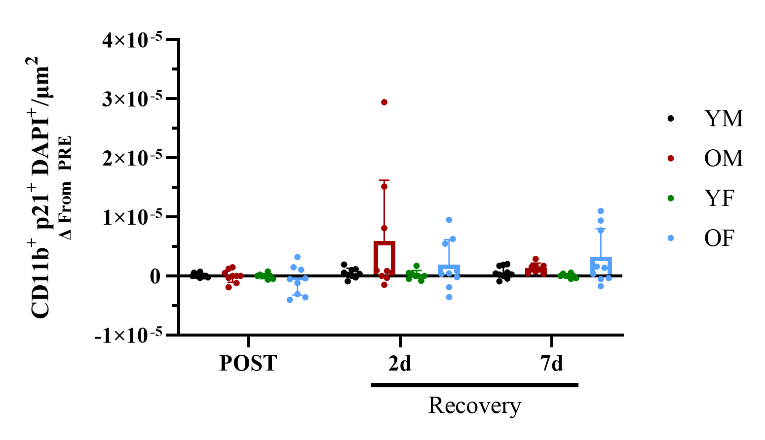


**Figure A6. Sex Differences in Cellular Senescence and Senescent Macrophages.**

**(A-C) Cellular Senescence (SA-β-Gal^+^)**: (A) Comparing young males and females in SA-β-Gal^+^ cells per µm^2^ (PRE: YA: n=17 (9M/8F), OA: n=18 (9M/9F); POST: YA: n=17 (9M/8F), OA: n=18 (9M/9F); 2d-Recovery: YA: n=16 (8M/8F), OA: n=18 (9M/9F); 7d-Recovery: YA: n=17 (9M/8F), OA: n=17 (8M/9F). (B) Comparing older males and females in SA-β-Gal^+^ cells per µm^2^. (C) Comparing sex differences in number of SA-β-Gal^+^ cells per µm^2^ (Δ) from PRE.

**(D-F) Senescent Macrophages (CD11b^+^ p21^+^ DAPI^+^)**: (A) Comparing young males and females in senescence macrophages per µm^2^ (PRE: YA: n=18 (10M/8F), OA: n=18 (9M/9F); POST: YA: n=18 (10M/8F), OA: n=18 (9M/9F); 2d-Recovery: YA: n=17 (9M/8F), OA: n=18 (9M/9F); 7d-Recovery: YA: n=18 (10M/8F), OA: n=17 (8M/9F). (B) Comparing older males and females in senescent macrophages per µm^2^. (C) Comparing sex differences in number of senescent macrophages per µm^2^ (Δ) from PRE.

Data±SD. OA, older adults; OF, older females; OM, older males; YA, young adults; YF, young females; YM, young males.


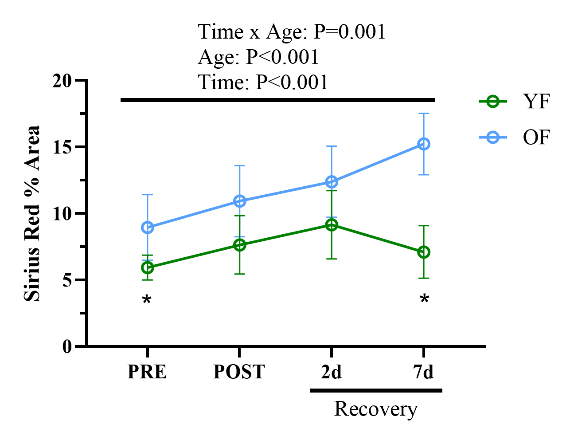

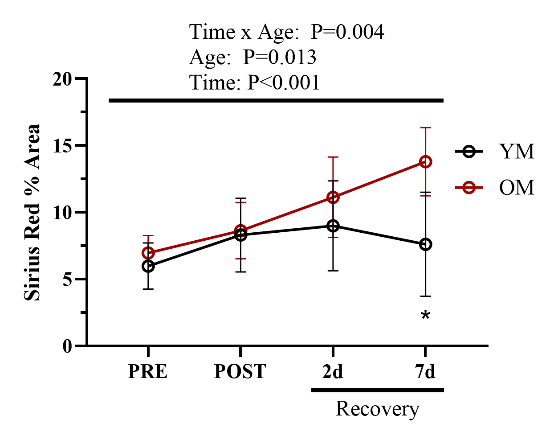


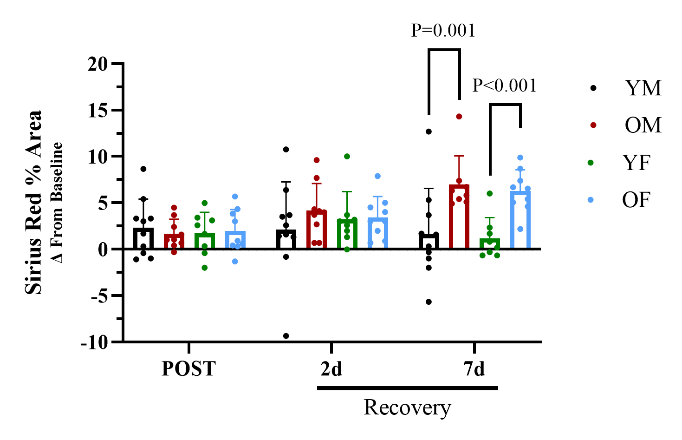


(C)

(A)

(B)

(A)

(A)

(A)

**Figure A7. Sex Differences in Collagen Content.**

**(A-C) Collagen Content (Sirius Red)**: (A) Comparing young males and females in % Sirius Red (PRE: YA: n=18 (10M/8F), OA: n=18 (9M/9F); POST: YA: n=18 (10M/8F), OA: n=18 (9M/9F); 2d-Recovery: YA: n=17 (9M/8F), OA: n=18 (9M/9F); 7d-Recovery: YA: n=18 (10M/8F), OA: n=17 (8M/9F). (B) Comparing older males and females in % Sirius Red. (C) Comparing sex differences in number of % Sirius Red (Δ) from PRE.

Data±SD. OA, older adults; OF, older females; OM, older males; YA, young adults; YF, young females; YM, young males

**METHODOLOGY**

**Histology**

Sections were dried (>1 hr) prior to staining.

*Myosin Heavy Chain Composition*

Sections were fixed in acetone for 15 min. Then sections were circled with a hydrophobic pen (Sigma-Aldrich cat. #402176-1EA). Afterwards, sections were washed (3x5 min in 1xTBS-Tween (0.05%), and blocked for 1 hr (10% goat serum, 2% BSA in 1xTBS). Then primary antibodies were placed on the sections and incubated overnight at 4°C (Anti-Laminin, 2E8 (1:150); Anti-MHC I, DHSB cat. #BA-D5 (1:200); Anti-MHC IIA, DHSB cat. #SC-71 (1:150). On the next day, sections were washed (4x5 min in 1xTBS-T) and then incubated with secondary antibodies for 1 hr (goat α-mouse (ThermoFisher AF488, IgG2a, 1:200); goat α-mouse (ThermoFisher AF555, IgG2b, 1:200); goat α-mouse (ThermoFisher AF647, IgG1, 1:200). Afterwards, sections were washed (3x5 min in 1xTBS-T) and coverslips were mounted on the slides with VectaShield Antifade mounting media (cat. #H-1000-10).

*Satellite Cells*

Sections were fixed in acetone for 7 min in -20°C and then traced with a hydrophobic pen. Afterwards, sections were washed (2x5 min in 1xPBS). Sections were blocked in 3% hydrogen peroxidases for 10 min. Then sections were washed (2x5 min in 1xPBS), and subsequently, covered in True Black (Biotium cat. #23007) which was removed immediately removed and washed (3x5 min in 1xPBS). Next, sections were blocked for 1 hr (5% goat serum, 2.5% horse serum, 0.1% triton with 1xPBS). Sections were washed (1x5 min) and then incubated in a streptavidin (Vector Labs cat. #SP-2002) solution (2.5% horse serum, 4 drops of streptavidin per 1 ml of 2.5% horse serum in 1xPBS). Again, sections were washed 1x5 min (1xPBS). Then sections were incubated with biotin (Vector Labs cat. #SP-2002) (2.5% horse serum, 4 drops of biotin per 1 ml of 2.5% horse serum in 1xPBS). Sections were washed for 1x2 min (1xPBS) then incubated with primary antibodies overnight at 4°C (PAX-7-supernatant, IgG1, 1:20; DHSB cat. #AB_528428). On the next day sections were washed (4x5 min in 1xPBS) and then incubated with Biotin (goat α mouse, IgG1, 1:1000; Jackson Immun: Cat. # 115-065-205) in 2.5% horse serum for 90 min. Next the samples were washed (3x5 min in 1xPBS) and then incubated with streptavidin (SA-HRP, 1:500; ThermoFisher cat. #S911). Again, sections were washed (3x5 min in 1xPBS) and then incubated with tyramide reagent superboost (TSA-555, 1:200; ThermoFisher cat. #B40923) for 20 min. After washing the slides (3x5 min in 1xPBS), sections were incubated with DAPI (1:000; Invitrogen, cat. #D3571) and 647 WGA (1:50) for 10 min. Lastly, slides were washed (2x2 min in 1xPBS) and coverslipped with VectaShield mounting media.

*Fibrosis (Sirius Red)*

Sections were fixed in Bouin’s Fixative (Sigma-Aldrich cat. #HT10132) for 1 hr at 56°C. Then samples were washed in DI water (3x5 min). Next sections were incubated with Picrosirius Red (abcam cat. #ab150681) for 40 min. Afterwards, samples were washed in 0.5% acetic acid (3x5 min) and then with DI water (3x5 min). Samples were then dehydrated by dipping the section in 90%, 95%, and 100% ethanol for 20 seconds each. Then sections were dipped quickly in xylene and placed coverslips on the slides with XYL mounting media (Electron Microscopy Siences cat. #18009).

*Hematoxylin & Eosin*

The samples were first fixated in 25% glutaraldehyde for 10 minutes and then washed in 1xPBS (1x3 min). Then samples were incubated with hematoxylin for 10 sec then immediately washed for 1x3 min (1xPBS). Afterwards, sections were incubated with eosin for 6 min. Then samples were dehydrated by dipping the section in 70, 95, and 100% ethanol for 5 sec each. Then immediately afterwards the samples were placed in xylene for 10 sec and then coverslipped with XYL mounting media.

*Fibro-Adipogenic Progenitors*

First, sections were circled with a hydrophobic pen and then fixed with 4% PFA for 7 min. Afterwards, samples were washed in 1xPBS (3x3 min). Sections were blocked for 1 hr with 2.5% horse serum in 1xPBS. Then the samples were incubated with primary antibody and WGA overnight (PDGFRα, goat, 1:100, R&D Systems cat. #AF-307-NA; WGA, AF488, 1:100). On the next day, samples were washed (3x5 min in 1xPBS) and were incubated with secondary antibodies (rabbit α goat AF555, 1:100; DAPI (1:1000). Then the samples were washed (3x5 min in 1xPBS) and coverslipped with VectaShield mounting media.

*Global Senescence (SA-β-Gal)*

First sections were fixed with 25% glutaraldehyde for 10 min. Afterwards, samples were washed with 1 x PBS (2x5 min) and stained with SA-β-Gal for 72 hrs at 37°C (Solution replaced every 24 hrs). The SA-β-Gal solutions contain 0.2M NA2HPO4/0.1M citric acid buffer (pH=6.0), 5M NaCl, 100mM K3Fe(CN)6, 100mM K4Fe(CN)6, 1M MgCl2, 20mg/ml X-Gal (Sigma-Aldrich cat. #B4252-100MG; Cell Signaling cat. #12767), and Distilled water. After 72 hrs, sections were washed in 1xPBS (3x5 min) and then finished with the H&E protocol that is described above.

*Senescent Macrophages*

Sections were fixed in acetone for 7 min in -20°C and then traced with a hydrophobic pen. Afterwards, sections were washed (2x5 min in 1xPBS). Sections were blocked in 3% hydrogen peroxidases for 10 min. Then sections were washed (2x5 min in 1xPBS). Sections were covered in True Black (Biotium) and then immediately removed and washed (3x5 min in 1xPBS). Next, sections were blocked for 1 hr (5% goat serum, 2.5% horse serum, 0.1% triton with 1xPBS). Sections were washed (1x5 min in 1xPBS) and then incubated in a streptavidin (Vector Labs) solution (2.5% horse serum, 4 drops of streptavidin per 1 ml of 2.5% horse serum in 1xPBS). Again, sections were washed 1x5 min (1xPBS). Then sections were incubated with biotin (Vector Labs) (2.5% horse serum, 4 drops of biotin per 1 ml of 2.5% horse serum in 1xPBS). Sections were washed for 1x2 min (1xPBS) then incubated with primary antibodies overnight at 4°C (CD11b, mouse IgG1, 1:100; Cell Sciences, cat. #MON1019-1). On the next day sections were washed (4x5 min in 1xPBS) and then incubated with Biotin (goat α mouse, IgG1, 1:1000; Jackson Immun: Cat. #115-065-205) in 2.5% horse serum for 90 min. Next the samples were washed (3x5 min in 1xPBS) and then incubated with streptavidin (SA-HRP, 1:500; ThermoFisher cat. #S911). Again, sections were washed (3x5 min in 1xPBS) and then incubated with tyramide reagent superboost (ThermoFisher TSA-555, 1:200) for 20 min. After washing the slides (3x5 min in 1xPBS), slides were reblocked for 1 hr (5% goat serum and 2.5% horse serum in 1xPBS). After washed (1x2 min in 1xPBS), sections were incubated in a streptavidin (Vector Labs) solution (2.5% horse serum, 4 drops of streptavidin per 1 ml of 2.5% horse serum in 1xPBS). Again, sections were washed 1x5 min (1xPBS). Then sections were incubated with biotin (Vector Labs) (2.5% horse serum, 4 drops of biotin per 1 ml of 2.5% horse serum in 1xPBS). Sections were washed for 1x2 min then incubated with primary antibodies overnight at 4°C (p21, 1:200; Cell Sciences, Abcam. #ab109199). On the next day, the sections were washed (4x5 min in 1xPBS) and then incubated with secondary antibody for 1 hr (goat α-rabbit, 1:1000, Jackson Immuno: Biotin-SP conjugated cat. #111-065-003). Afterwards, samples were washed (3x5 min in 1xPBS) and then incubated with streptavidin conjugate for 1 hr (streptavidin, SA-AF488, 1:500, Thermo cat. #S32354). Then sections are washed (3x5 min in 1xPBS) and then incubated with DAPI (1:000; Invitrogen, cat. #D3571) and 647 WGA for 10 min (1:50). Lastly, slides were washed (2x2 min in 1xPBS) and coverslipped with VectaShield mounting media.

*Macrophages*

Sections are fixed in chilled acetone for 3 min and then washed in 1xPBS (3x3 min). Afterwards, slides are blocked with 3% endogenous peroxidases in 1xPBS for 8 min. Then samples are washed (3x3 min) and then blocked for 1 hr (2.5% horse serum with vector avidin D solution (4 drops per 1 ml solution, Vector cat. #S-2012). Next, slides are incubated with primary antibody for 1 hr (cd68, 1:100, ThermoFisher cat. #14-068-82). Afterwards, sections were washed (4x5 min in 1xPBS) and then secondary antibodies are applied to the samples for 1 hr (donkey α-mouse IgG Biotin – SP conjugated, 1:500, Jackson Immuno cat. #715-065-150). Next samples are washed (4x5 min in 1xPBS) and then incubated with SH-HRP (1:500, Invirogen cat. #S-911) for 30 min. The samples were then washed (3x5 min in 1xPBS) and tyramide reagent superboost (ThermoFisher AF488, 1:500) was applied to the samples for 10 min. Sections were washed (3x3 min in 1xPBS) and then reblocked with the same blocking solution for 10 min. Then samples were washed (1x2 min in 1xPBS) and then incubated with primary antibody (cd206, 1:200, R&D cat. #AF2534) overnight at 4°C. On the next day, slides were washed (4x5 min in 1xPBS) and sections were incubated with secondary antibody for 1 hr (bovine α-goat, Cy3, 1:250, Jackson Immuno cat. #805-165-180). Next, samples were washed (3x5 min in 1xPBS) and then incubated with 647 WGA (1:50) and DAPI (1:1000) for 10 min. Lastly, samples are washed (2x2 min in 1xPBS) and coverslipped with vector shield mounting media.

*MPO and Dystrophin*

First, sections were fixed with 4% PFA for 5 min and then circled with a hydrophobic pen. Samples were then washed (2x10 min in 1xPBS-Tween (0.05%). Samples were blocked for 45 min (1% BSA in 1xPBS) and then incubated with primary antibodies overnight (Laminin: 1:2000, DHSB cat. #2E8; MPO: 1:2000 (GeneTex cat. #GTX135125) or Dystrophin: 1:100 (abcam cat. #AB15277). On the next day, slides were washed (3x10 min in 1xPBS-T) and then incubated with secondary antibodies (goat α-rabbit 594, 1:200; goat α-mouse 488, 1:200). Afterwards, sections were washed (3x10 min in 1xPBS-T) and then incubated with DAPI (1:1000) for 10 min. Lastly, slides are washed (1x2 min in 1xPBS) and coverslipped with VectaShield mounting media.

*CD45 & CD66b*

First, sections were fixed in chilled acetone for 10 minutes and then washed for 5 minutes in PBS. Next, section were covered in True Black (Biotium) and then immediately removed and washed (3x5 min in 1xPBS). Sections were blocked with 5% goat serum in 1xPBS for one hour and later incubated with 1° antibody (CD66b, Thermo cat. #MAG-51806 (1:100)) with 5% goat serum overnight at 4°C. On the next day, samples are washed (4x5 min in 1 xPBS) and then incubated with goat anti-mouse IgG1 2° antibody (Thermo cat. #A-21121 (1:100)) for 1 hour. Afterwards, sections are washed (3x5 min in 1xPBS) and then reblocked with 5% goat serum in 1xPBS for 1 hour. Next, samples are incubated with 1° antibody (CD45, ABCAM cat. #ab30470 (1:100)) for 1 hour. Sections are then washed (4x5 min in 1xPBS) and then incubated with goat anti-mous3 IgG2a (Thermo cat. #SA5-10370-AFP555) for 1 hour. Afterwards, samples are washed (3x5 min in 1xPBS) and then incubated with 647 WGA (1:50) and DAPI (1:1000) for 10 minutes. After the samples are washed (2x5 min in 1xPBS) slides are coverslipped with VectaShield mounting media.
